# Exposure to Indoor Allergens in Different Residential Settings and Its Influence on IgE Sensitization in a Geographically Confined Austrian Cohort

**DOI:** 10.1371/journal.pone.0168686

**Published:** 2017-01-03

**Authors:** Teresa Stemeseder, Bettina Schweidler, Patrick Doppler, Eva Klinglmayr, Stephanie Moser, Lisa Lueftenegger, Martin Himly, Roland Lang, Joerg Zumbach, Gertie J. Oostingh, Thomas Hawranek, Arne C. Bathke, Gabriele Gadermaier

**Affiliations:** 1 Department of Molecular Biology, University of Salzburg, Salzburg, Austria; 2 Department of Mathematics, University of Salzburg, Salzburg, Austria; 3 School of Education, University of Salzburg, Salzburg, Austria; 4 TUM School of Education, Technical University of Munich, Munich, Germany; 5 Department of Dermatology, Paracelsus Medical University Salzburg, Salzburg, Austria; 6 Biomedical Sciences, Salzburg University of Applied Sciences, Puch/Salzburg, Austria; Telethon Institute for Child Health Research, AUSTRALIA

## Abstract

**Background:**

Exposure to indoor allergens is crucial for IgE sensitization and development of allergic symptoms. Residential settings influence the allergen amount in house dust and hence allergic sensitization. Within this study, we investigated allergen exposure and molecule-based IgE levels in a geographically confined region and evaluated the impact of housing, pets and cleaning.

**Methods:**

501 adolescents from Salzburg, Austria participated in this cross-sectional study. House dust samples were examined regarding major mite, cat, dog, and mold allergens using a multiplex assay. Serum samples of participants were analyzed for specific IgE to Der p 1, Der p 2, Fel d 1, Can f 1 and Alt a 1 using the multiplex array ImmunoCAP ISAC. Information on allergies, living areas, dwelling form (house, flat, farm), pets, and household cleanliness were obtained by a questionnaire.

**Results:**

In investigated house dust samples, the concentration of cat allergen was highest while the prevalence of mold allergens was very low. Participants showed IgE sensitization to Der p 1 (13.2%), Der p 2 (18.2%), Fel d 1 (14.4%), Can f 1 (2.4%) and Alt a 1 (2.0%). In alpine regions, lower mite allergen concentrations were detected which correlated with reduced IgE levels. A trend for increased sensitization prevalence from rural to alpine to urban regions was noted. Living on farms resulted in lower sensitization prevalence to mite and cat allergens, even though exposure to mites was significantly elevated. The presence of cats was associated with a lower sensitization rate and IgE levels to cat and mite allergens, and less frequent allergic diseases. Cleaning did not impact allergen concentrations, while IgE reactivity to mites and allergic diseases were more pronounced when living in cleaner homes.

**Conclusion:**

Allergen exposure to indoor allergens was influenced by setting of homes. Living in a farm environment and having a cat at home showed a protective effect for IgE sensitization and allergies. This cross-sectional study in combination with hereditary and lifestyle factors enables development of risk schemes for a more efficient management and potential prevention of allergic diseases.

## Introduction

Exposure to house dust is one of the basic features for the development of allergic symptoms to inhalant indoor allergens [1, 2]. The most common allergens found in house dust originate from mites, animal dander, molds and cockroaches [3]. Major allergens typically represent the most relevant IgE-binding molecules of an allergen source and are involved in triggering allergic symptoms [4]. The dominant allergens of the most common house dust mite species (*Dermatophagoides pteronyssinus* and *Dermatophagoides farinae)* belong to mite group 1 (Der p 1 and Der f 1) and mite group 2 (Der p 2 and Der f 2) allergens. These molecules account for IgE sensitization in more than 80% of mite-allergic subjects and a high level of cross-reactivity between mite species exists [3].

Fel d 1 is the major allergen from cat (*Felis domesticus*) and more than 80% of the total IgE reactivity to cat allergens is directed against Fel d 1 [5]. It accounts for a large proportion of allergens from animal dander in house dust, but large variations in allergen concentrations have been noted in households of different countries [6, 7]. The second most important animal allergen in house dust is Can f 1 from dog (*Canis familiaris*) with a sensitization prevalence of 75% among dog-allergic patients [8]. Similar to cat allergens, Can f 1 is frequently found in European households [6].

*Alternaria alternata* represents one of the most common molds and its major allergen Alt a 1 is recognized by >90% of *Alternaria*-sensitized patients [9, 10]. Due to perennial mold exposure and the association with asthma and respiratory allergies, investigating the relevance of exposure and IgE sensitization is of special interest [3, 6]. Allergen exposure to cockroach can be high in inner-city areas of metropolises, while very low levels were found in Central Europe due to a mostly suboptimal habitat for these insects [11, 12].

Exposure to allergens is a prerequisite for initiating an allergic sensitization leading to the production of allergen-specific IgE antibodies [13]. Exposure to indoor allergens has been linked to IgE sensitization and development of allergic symptoms in a number of studies. However, there are controversial findings regarding the kind of correlation, ranging from a protective effect upon high exposure to certain allergens [14–16] to a negatively influencing effect [17–19], whereas other studies found no effect at all [20, 21]. Also, exposure itself is influenced by different factors such as pet ownership, infrastructural characteristics or altitude. These influences are widely discussed in the literature and strong differences in exposure between groups [6, 21–23] as well as no effects [24] from specific influencing factors are found. In order to assess allergen exposure, it is well established to measure allergen concentrations in house dust samples using antibody-based detection systems [3]. Usually, 2–4 sites within a household are sampled for the assessment of allergen exposure, typically including mattresses, bedding, carpets, and sofas [2, 25]. Since IgE antibodies are a prerequisite for the development of allergies, linking IgE antibodies with allergen exposure in households can provide valuable information regarding risk factors and to suggest prevention measurements, thus contributing to diminish the progress of allergic diseases [26]. So far, no study investigating the association between indoor allergen exposure and IgE sensitization to a panel of purified allergen molecules in a large non-selected cohort is available.

The aim of this study was to analyze the exposure and IgE sensitization to five major indoor allergens originating from mite, cat, dog and mold in samples from different residential settings. Therefore, allergen concentrations of Der p 1, mite group 2, Fel d 1, Can f 1 and Alt a 1 were measured in house dust of homes. IgE sensitization to corresponding purified allergens was analyzed in sera of 501 adolescents and the relevance of different living areas, dwelling forms, pets or household cleanliness was evaluated. Allergen exposure was also evaluated with respect to reported allergic diseases.

## Methods

### Study design and participants

This cross-sectional study was conducted in a non-selected cohort of 501 pupils from schools in different geographic regions in the district of Salzburg, Austria. Participants were recruited from school grades 8–13 (expected age 13–19 years) and sampling took place in the time between October 2013 and May 2014. Study enrollment and demographic data of participants are presented in Tables 1 and 2; further details can be found in Stemeseder *et al*. [27]. Written informed consents from the participating subjects themselves and their legal guardian (if they were younger than 18 years) were obtained. The study was conducted according to common ethical principles and approved by the local ethics committee of Salzburg, Austria, No. 415-E/1669/6-2013.

**Table 1:** Study enrollment.

Total contacted n = 659	Agreed to participate n = 545	Informed consent received n = 523	Final study group n = 501
	**Declined to participate** n = 114	**Informed consent not received** n = 22	**Absent for data collection** n = 22

**Table 2:** Demographic data of participants.

	Mean (range) or % (n/N)		
	Female (n = 279)	Male (n = 222)	Total (n = 501)
Age (years)	15.3 (13–21)	15.1 (12–20)	15.2 (12–21)
Atopic individuals (%)	50.9 (142/279)	56.8 (126/222)	53.5 (268/501)
Clinically diagnosed allergy (%)	21.7 (56/258)	22.1 (46/208)	21.9 (102/466)
Self-reported allergy (%)	47.1 (115/244)	40.9 (74/181)	44.5 (189/425)

### Assessment of allergen exposure in house dust samples

Individual house dust samples were collected by study participants using a commercially available DUSTREAM Collector (Indoor Biotechnologies, Charlottesville, VA, USA) attached to a household vacuum cleaner. They followed the detailed protocol from Indoor Biotechnologies and were additionally instructed and trained by the allergy research team. Four areas each sized 20 x 30 cm on mattress (head and foot area), bedroom carpet and living room couch were sampled for 30 s per area. Dust extracts were prepared by dissolving 100 mg of fine dust in 2 ml of phosphate-buffered saline pH 7.4, 0.05% Tween-20 (PBS-T). Samples <10 mg of fine dust were not considered for further analysis. Proteins were extracted by shaking for 2 h at room temperature following a centrifugation step at 1,380 x *g* for 20 min. Supernatants were stored at -20°C until further processing.

For allergen content analysis, the dust extracts were thawed and centrifuged again. Supernatants were diluted 1:10, 1:100 and 1:10,000 and examined in a Multiplex Array for Indoor Allergens (MARIA, Indoor Biotechnologies, Charlottesville, VA, USA) using xMAP Technology (Luminex, Austin, TX, USA) [28]. The array uses fluorescently labeled beads conjugated to monoclonal antibodies specific for purified allergen molecules. The allergen concentration of Der p 1 and mite group 2 allergens (house dust mites allergens), Fel d 1 (cat allergen), Can f 1 (dog allergen) and Alt a 1 (mold allergen) was investigated. A 12-point standard curve executed in duplicates was used to quantify the results. Additionally, quality controls provided with the test kit were applied. Measurements of fluorescence were performed in a Luminex200IS (Luminex, Austin, TX, USA) with Luminex100IS software (build 2.3). Raw data were imported to Masterplex QT v4.0 software (Hitachi Solutions America Ltd., San Bruno, CA, USA) for further analysis. Lower limit of detection (LLOD) was set to 3 x SD + mean of blank values and the standard curve was calculated using a five parameter logistics curve fit. Concentrations of allergens were calculated as mean of the three dilution-adjusted measurements per sample. Sample values with a bead count lower than 50 beads per analyte as well as values below the LLOD were excluded for mean calculation.

### Blood sampling and specific IgE analysis using an allergen multiplex array

Capillary blood samples were obtained from the fingertip and incubated at room temperature for 15 min. After centrifugation at 14,000 rpm, serum was separated from the blood cells. Serum samples were subsequently stored at 4°C for transport and -20°C until further analysis. Analysis of sera for specific IgE to single purified allergens was done by means of the allergen multiplex array ImmunoCAP ISAC (Thermo Fisher Scientific, Uppsala, Sweden). According to the manufacturer’s protocol, the test was performed with 30 μl of serum (Protocol No. 20-01-02-6). The resulting fluorescent signals were measured with a confocal laser scanner (LuxScan-10K, CapitalBio, Beijing, China). Data were analyzed using Phadia Microarray Image Analyzer (MIA) software and transformed into semi-quantitative ISAC Standardized Units (ISU). Specific IgE values ≥0.3 ISU were considered positive. Participants with a positive value to any of the 112 allergens on the ImmunoCAP ISAC were considered as sensitized.

### Assessment of personal and demographic data

Participating pupils filled out an in-house developed written questionnaire which was anonymous and linked to the IgE data and the dust sample using a number code. Demographic data such as gender and age were gathered. Furthermore, subjects reported on their living area *i*.*e*. urban (city of Salzburg), rural, or alpine (>800 m above sea level), their dwelling form *i*. *e*. house, flat, or farm, and the presence of pets within their homes. Additionally, they reported on self-assessed household cleanliness considering frequency of mattress and bedsheet exchange as well as vacuum cleaning and comparison of their homes to a very sterile environment. Cleanliness was assessed by values ranging from 1 to 5, with 5 indicating the highest cleanliness. All study participants were asked to report if they suffer from any allergy which was diagnostically confirmed by a clinician.

### Statistical analysis

In order to detect a nonparametric relative effect of 0.65 with the two-sided two-sample rank sum “Mann-Whitney” test, the approximate minimal sample size necessary to obtain power = 0.9 at alpha = 0.05 is n = 61 per group, using Noether’s formula. This sample size was always exceeded in our pairwise comparisons, where the smallest group had n = 71. Statistical analysis was performed with R in RStudio [29] and GraphPad Prism 5 for Windows (GraphPad Software, Inc., La Jolla, CA, USA). Correlations between ISU levels and allergen concentrations in house dust were calculated as Spearman’s rank correlation. Comparisons of ISU levels or allergen concentrations between groups were performed using Mann-Whitney tests. Odd’s ratios were calculated from Fisher’s exact test for count data. P-values <0.05 were considered as statistically significant. P-values are reported without multiplicity adjustment throughout the manuscript and were categorized as follows: p<0.05 (*), p<0.01 (**), p<0.001 (***), p<0.0001 (****).

## Results

### Study cohort

501 pupils participated in the study by donating blood samples and returning the questionnaire. Dust samples from 96.0% of participants were considered for further analysis. Of 501 pupils, 71 were living in an urban region, 264 in rural regions and 165 in alpine regions. Participants also stated in which kind of dwelling they lived: 113 lived in flats, 310 in houses and 74 on a farm. Regarding pets, 340 (67.9%) reported having pets, 236 (47.1%) had a cat and 88 (17.6%) had a dog at home. Self-assessed household cleanliness was reported with a mean value of 2.94 assessed on a scale from 1 to 5, where 1 corresponded to very low cleanliness and 5 was highest cleanliness.

### Indoor allergen exposure

**Fig 1:**
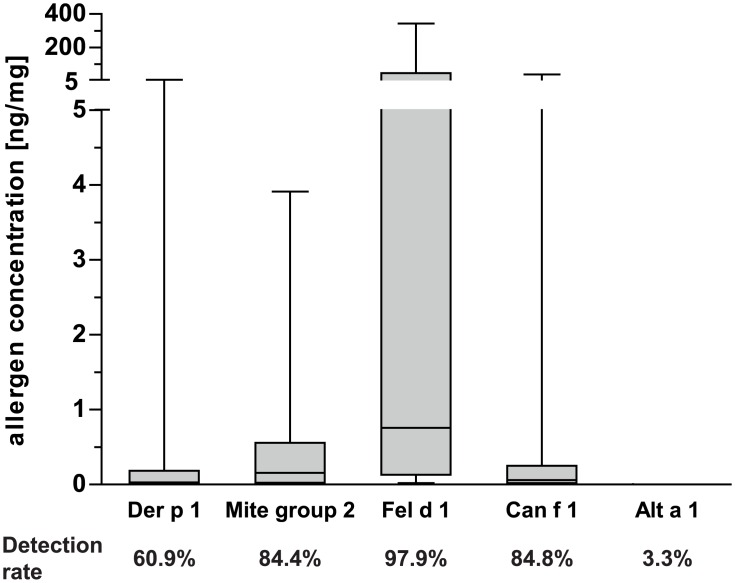
Allergen concentrations found in investigated house dust samples and respective detection rates. Boxes indicate 25^th^ to 75^th^ percentile, horizontal line represents median, whiskers indicate 5^th^ and 95^th^ percentiles.

Indoor allergen concentrations in house dust samples were measured by the MARIA system (Fig 1). The major cat allergen Fel d 1 was detected in 97.9% of investigated homes. It represented the predominant allergen (median 0.76 ng/mg fine dust) and reached values >343.7 ng/mg fine dust in 5% of the homes. Median concentrations of mite allergen Der p 1, mite group 2 and dog allergen Can f 1 were 0.03 ng/mg, 0.16 ng/mg and 0.06 ng/mg, respectively. Alt a 1 was detected in 3.3% of homes only and allergen concentrations were insignificantly low and thus not considered for further correlation analyses. Concentrations of house dust mite allergens (Der p 1 and mite group 2) were highly correlating in the samples (p<0.0001, rho = 0.7). Notably, mite group 2 allergens were detected more frequently and at higher concentrations than Der p 1. A slight correlation (p<0.0001, rho = 0.2) was also found for Can f 1 and Fel d 1. No significant correlations were found between other allergens.

**Fig 2:**
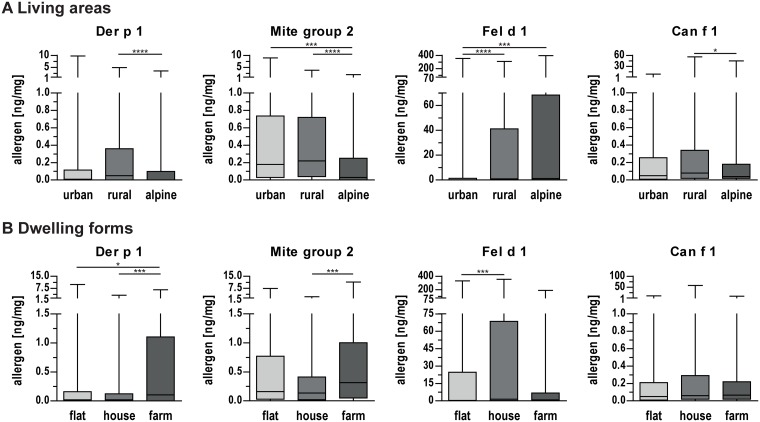
Allergen concentrations in house dust samples collected in different housing settings. **(A)** Allergen concentrations in different living areas (urban, rural and alpine). **(B)** Allergen concentrations in different dwelling forms (flat, house and farm). Boxes indicate 25^th^ to 75^th^ percentile, horizontal line represents median, whiskers indicate 5^th^ and 95^th^ percentiles. *, p<0.05; ***, p<0.001; ****, p<0.0001 for pairwise comparisons.

The concentrations of indoor allergens were additionally analyzed with respect to different living areas (Fig 2A). Considerably lower concentrations of mite allergen Der p 1 and mite group 2 allergens were found in alpine households. Cat allergen Fel d 1 was found to be less prevalent in urban homes in comparison to rural and alpine homes. Lower concentrations of Can f 1 were identified in alpine homes. Likewise, we observed variances in different forms of dwellings, *i*. *e*. flats, houses and farms (Fig 2B). Increased concentrations of mite allergens Der p 1 and mite group 2 were found in house dust samples of farms. The concentration of cat allergen Fel d 1 was higher in houses compared to flats or farms while no significant difference could be found for the dog allergen Can f 1.

**Fig 3:**
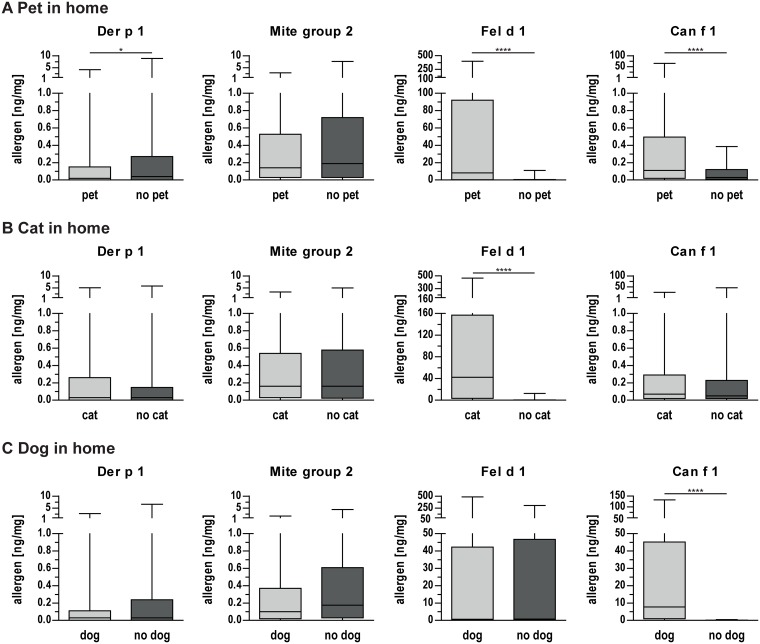
Allergen concentrations in house dust samples collected in households with and without pets. **(A)** Allergen concentrations in homes with and without a pet. **(B)** Allergen concentrations in homes with and without a cat. **(C)** Allergen concentrations in homes with and without a dog. Boxes indicate 25^th^ to 75^th^ percentile, horizontal line represents median, whiskers indicate 5^th^ and 95^th^ percentiles. *, p<0.05; ****, p<0.0001 for pairwise comparisons.

Differences in allergen concentrations were also identified between homes with and without pets. In house dust samples of participants with a pet at home, lower concentrations of mite allergen Der p 1 but higher concentrations of Fel d 1 and Can f 1 were found (Fig 3A). In house dust of cat owners, significantly higher concentrations of Fel d 1 were found (Fig 3B). Likewise, in homes of dog owners, considerably higher concentrations of Can f 1 were present (Fig 3C). No statistically significant differences were found for other allergen concentrations with respect to pet, cat, or dog present at home. No correlation of allergen concentration and self-reported household cleanliness was found for any of the investigated allergens.

### IgE sensitization to indoor allergens

**Fig 4:**
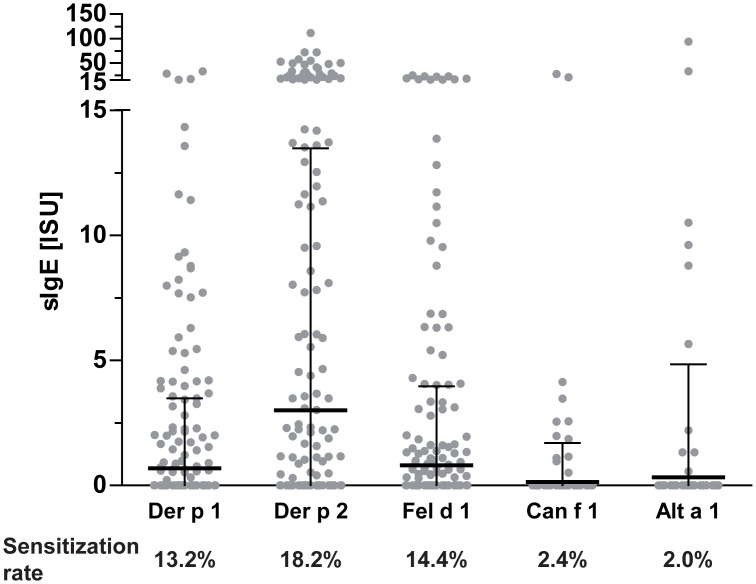
Dot plot of the specific IgE levels to indoor allergens and respective sensitization rates. Dots represent individual measurements, lines indicate mean values and whiskers the standard deviation.

IgE sensitization to indoor allergen molecules was evaluated by means of allergen multiplex array ImmunoCAP ISAC. As we have previously reported, a general sensitization rate of 53.5% was found in the study population [27]. IgE levels of the mite group 2 allergens Der p 2 and Der f 2 were highly similar and strongly correlating (p<0.0001, rho = 0.99), therefore Der p 2 was used for subsequent statistical analyses. IgE sensitization rates ranged from 13.2% to 18.2% for mite allergens Der p 1 and Der p 2 and cat allergen Fel d 1 (Fig 4). Low sensitization rates were detected for Can f 1 (2.4%) as well as for the mold allergen Alt a 1 (2.0%). Highest IgE levels were found for the mite allergen Der p 2 (mean: 3.0 ISU). Although the sensitization prevalence was very low, some Alt a 1-positive individuals presented rather high IgE levels.

Weak but statistically significant positive correlations between allergen concentrations in house dust and IgE levels were found for mite group 2 allergen concentration and IgE levels to Der p 1 (p<0.05, rho = 0.11) as well as for Can f 1 dog allergen concentration and Can f 1 IgE levels (p<0.05, rho = 0.11). Interestingly, concentrations of Fel d 1 showed a slightly negative correlation with IgE levels to Der p 2 (p<0.05, rho = -0.12).

### Residential settings and their impact on IgE sensitization

**Fig 5:**
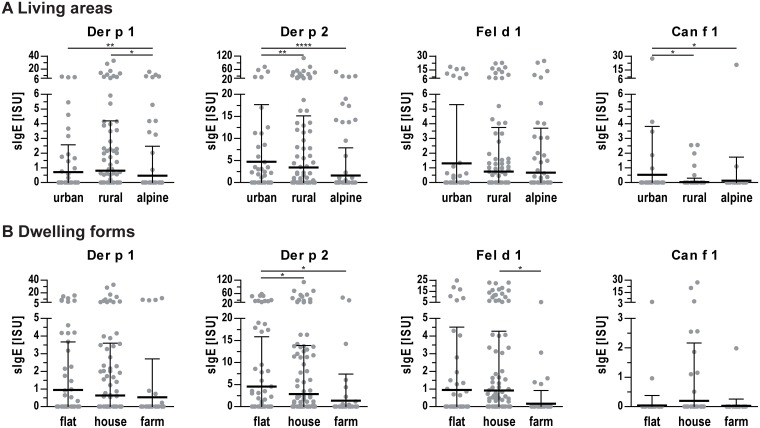
IgE levels to indoor allergens in sera of pupils living in different settings. **(A)** IgE levels of pupils living in different areas (urban, rural, alpine). **(B)** IgE levels of pupils living in different dwelling forms (flat, house, farm). Dots represent individual measurements, lines indicate mean values and whiskers the standard deviation. *, p<0.05; **, p<0.01; ****, p<0.0001.

Potential influences of different residential settings on IgE sensitization were investigated. The overall sensitization rate did not differ significantly between the three investigated living areas but a trend of increased sensitization from rural (51.5% sensitized) to alpine (55.2%) to urban (57.7%) regions was observed. However, pupils living in alpine areas showed a significantly decreased sensitization prevalence and lower IgE levels to mite allergens Der p 1 and Der p 2 (Fig 5A). No statistically significant difference between living areas was found for Fel d 1. Increased Can f 1 IgE levels were observed for pupils living in urban regions, but case numbers were rather low.

With regard to different dwelling forms, a significant difference (p<0.05) was found in the overall sensitization rate with an increase from farm (41.9% sensitized) to house (53.9%) to flat (60.2%). Living on a farm (74 subjects) was associated with a decreased odd’s ratio of 0.577 (95% CI: 0.337–0.978, p<0.05) for general IgE sensitization. Pupils living in flats showed a higher sensitization prevalence and IgE levels to the mite allergen Der p 2 (Fig 5B). Pupils living on farms showed decreased prevalence and IgE levels to Fel d 1 compared to pupils living in houses.

**Fig 6:**
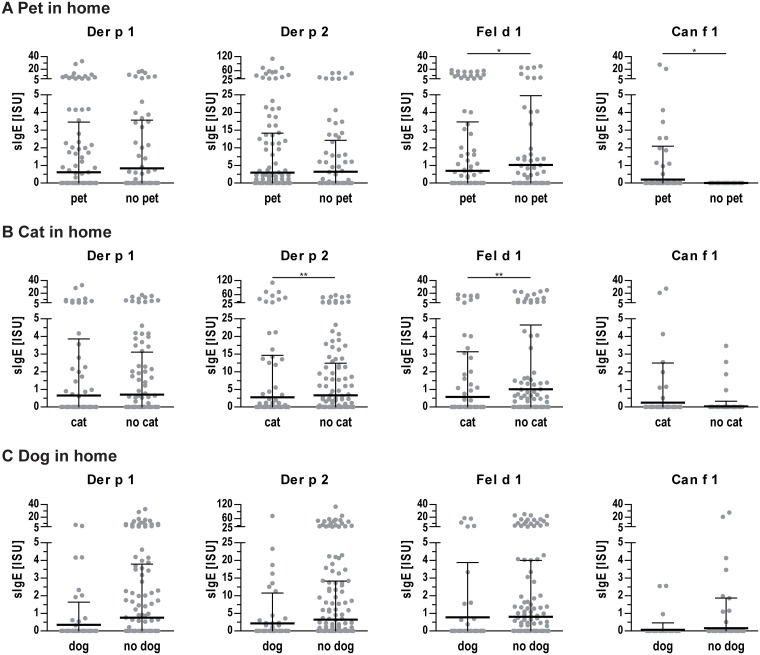
IgE levels to indoor allergens of participants with or without pets at home. **(A)** IgE levels of participants with or without any pet at home. **(B)** IgE levels of participants with or without a cat at home. **(C)** IgE levels of participants with or without a dog at home. Dots represent individual measurements, lines indicate mean values and whiskers the standard deviation. *, p<0.05; **, p<0.01.

Generally, a slightly lower sensitization rate of 52.4% was found for pupils living with any pet at home compared to 55.5% for those without a pet. Decreased IgE levels to Fel d 1 but slightly higher IgE levels to Can f 1 were found in homes of pet owners (Fig 6A). For cat owners, a significantly decreased general sensitization rate of 47.9% was found (p<0.05) which also translated into a decreased odd’s ratio of 0.658 (95% CI: 0.453–0.952, p<0.05). In addition, IgE levels to Der p 2 and Fel d 1 were lower for cat owners (Fig 6B). No differences in sensitization rates or IgE levels to single allergens were found between dog owners and non-dog owners (Fig 6C). A slight positive correlation was identified between self-reported household cleanliness and IgE levels to mite allergen Der p 1 (p<0.01, rho = 0.12); no statistically significant association was observed with other allergens.

### Residential settings and reported allergic diseases

Within the study population, 21.8% of participants reported to suffer from any allergy which was diagnosed by a medical doctor. Thus, allergen exposure was additionally analyzed in relation to a reported allergic disease. Interestingly, significantly decreased concentrations of Der p 1 mite allergen concentration were observed for allergic pupils compared to those who did not have any allergic disease (p<0.01).

With respect to living areas, a trend for a higher prevalence of reported allergic diseases was found for urban regions (25.4%) compared to rural (21.6%) and alpine regions (21.0%). For different dwelling forms however, a significantly decreased (p<0.01) rate of allergies was found among participants living on farms (9.6%), compared to higher rates for participants living in flats (28.2%) or houses (22.7%). While no difference in allergy prevalence could be found whether pupils had a pet at home or not, a slightly decreased but statistically not significant rate was found for cat owners (18.6% for cat owners *vs*. 24.3% for non-cat owners). For dog owners on the other hand, the rate of reported allergies was slightly increased with a diagnosis rate of 25.0% as compared to non-dog owners (20.7%). Interestingly, allergic pupils reported to live in a cleaner environment compared to those without symptoms (p<0.0001).

## Discussion

This cross-sectional study represents the first investigating exposure and molecule-based IgE sensitization to 5 major indoor allergens within a geographically confined area of Central Europe. Dust sampling and detection of indoor allergens as well as participants’ specific IgE levels was conducted with commercially available multiplex arrays, thus being highly reproducible and also comparable with other studies [27, 28]. Results showed that particularly cat allergen exposure, living on farms or at higher altitudes as well as household cleaning were significant parameters for IgE sensitization in our study cohort.

### Indoor allergen exposure and effect on IgE sensitization

Major allergens from cat, house dust mite, and dog were detected in more than 84% of investigated homes. We found particularly high concentrations of Fel d 1, while concentrations of Can f 1 were significantly lower which probably reflects households with 47.1% of cats but only 17.6% of dogs in our cohort. However, we noticed a slight correlation of Fel d 1 and Can f 1 which might reflect the general environmental abundance of these allergens [6]. Though highly correlating concentrations of mite group 1 and 2 allergens were observed, group 2 allergens were more prevalent in our cohort similar to a multicenter study using the same multiplex array technology [28, 30]. Within this study, Alt a 1 exposure was for the first time evaluated in a cross-sectional approach; concentrations were however low and thus not eligible for subsequent analysis. Using Alt a 1 as sole marker might be misleading since previously insufficient allergen release due to suboptimal growth conditions was observed [31]. Generally, recent changes in constructions of buildings might favor the increase of molds and fungi-related allergies [32].

Similar to previous studies we found high IgE sensitization rates to Der p 1 (13.2%), Der p 2 (18.2%) and Fel d 1 (14.4%) [33–35]. Can f 1 was tested positive in only 2.4% of sera while higher frequencies of 5% and 16.2%, respectively were found in other cohorts [34, 35]. The low prevalence of Alt a 1 sensitization (2%) in our study correlates well with skin prick test results in a Swedish cohort of 16–30 years olds [34], while sensitization in US was found to be almost 8%. Varying environmental conditions or divergent IgE test methods using extracts or single molecules may account for divergent findings, while positive individuals were shown to be clearly at risk for development of allergic reactions [35, 36].

Within this study, we observed a positive correlation between exposure to mite group 2 allergens and sensitization to Der p 1. Previous studies have shown an increased risk for dust mite sensitization with increasing Der p 1 exposure [37–39]. On the other hand, an intermediate level of mite allergen exposure was found to translate into highest sensitization rates [40]. Analogous, sensitization to pets was highest at moderate exposure levels [37], while we and others detected a positive correlation between Can f 1 exposure and sensitization [41]. Participants reporting to suffer from allergies showed lower exposure to Der p 1 in our study. We thus conclude that allergen exposure and subsequent development of IgE is triggered by the allergenic molecules at certain levels but highly influenced by other intrinsic and external factors relevant for the immune system [1].

### Living area

Different residential settings have been shown to play a role in allergy and thus urban, rural, and alpine regions in Salzburg were studied. Mite allergens were slightly more prevalent in rural areas, but differences were not as pronounced as in a Polish study revealing significantly higher exposure in rural regions [21]. We, however, found significantly reduced mite allergen concentrations in alpine regions, which also translated into reduced IgE levels. These findings correlate well with other studies showing decreased exposure in high altitudes [42, 43], and lower sensitization prevalence in alpine or dry regions [44, 45]. Interestingly, a recent study did not find a correlation between altitude and mite allergens in alpine regions of Germany and Austria but also noted lower Der f 1 levels at elevated altitudes [24]. Divergent outcomes may be due to investigations of homes as well as taverns and mountain huts in the latter study. Also various climatic conditions at high altitudes do not allow for absolute generalization of this finding [46].

We found a decreased concentration of cat allergen in urban homes while those study participants showed a trend for higher IgE levels to cat allergens similar to a study by Elhom *et al*. [47]. In our study, 22.5% of urban homes but >50% of investigated homes in rural or alpine regions have a cat at home [48]. Those findings seem to further underline the discussed protective effect of cat exposure [37, 47, 49]. No such protective effect was observed upon dog exposure in our study. In general, a trend for higher sensitization rates in urban regions was found supporting data showing 10, 20 and 40 percentage points higher sensitization rates compared to rural areas in 3 studies [33, 47, 50]. This was also reflected by a lower number of allergies reported from the rural population which might however be influenced by socioeconomic factors including consultation of medical care. The urban environment thus seems to be a risk factor for allergic diseases and might be particularly influential during childhood and adolescence.

### Dwelling forms

Significantly higher concentrations of mite allergens were determined in farm houses, while IgE levels to Der p 2 were significantly decreased in these study participants. High mite allergen exposure on farms was previously observed and might be linked to storage mites [38, 51, 52]. Interestingly, the PARSIFAL study conducted in 5 European countries did not find differences in group 1 allergen levels, while lower sensitization rates in farm children were noted [40]. Indoor exposure to cat allergens was lower in farms compared to houses probably relating to the fact that farming cats spend most of the time outside. Farm-living participants showed decreased IgE levels to Fel d 1, supporting findings from the afore-mentioned cross-sectional study [40]. Dog allergen exposure and IgE concentrations were not different in the investigated dwelling forms.

Analogous to previous studies, we also observed decreased allergy prevalence in participants living in farms [40, 53]. In particular, children with regular exposure to the farming environment were shown to have the lowest prevalence rates of wheezing and atopic diseases [54]. As a tendency for increasing IgE levels from farm to house to flat was observed, one might speculate that participants living in houses have more contact to farming environments but may benefit from green areas which have also been shown to have an allergy-protective effect [54, 55].

### Pets

Regarding pets, we found significantly higher concentrations of cat (248-fold) and dog allergens (193-fold) when respective animals were present at homes [6, 39, 56]. In our study cohort, a high presence rate of cats (47.1%) and dogs (17.8%) was noted, significantly higher compared to a European birth-cohort study with 7.2–35% (average 14.9%) cats and 5.4.-35% (average 12%) dogs in homes [57].

Especially cat ownership seemed to have a protective effect, indicated by lower total sensitization rates and reduced IgE levels to cat and mite allergens as well as slightly reduced allergy prevalence. Stratification by different living areas also revealed a decreased sensitization rate for cat owners in all investigated regions, but statistical significance was only achieved for living in rural areas. Especially living with cats was found to have an inverse relationship of allergic sensitization to cats and dogs, while no such impact was found for mites or grass pollen sensitization [37, 49]. There is however also data available suggesting that pet exposure does not influence or even poses an increased risk for IgE sensitization [20, 58]. Our results clearly indicate that actual exposure to cats is associated with lower IgE sensitization in the investigated adolescents, but overlapping protective effects originating from living in rural areas cannot be fully excluded.

### Household cleanliness

In our cohort, no correlation between allergen occurrence in the dust samples and self-estimated cleanliness of homes could be verified. Although reported household cleanliness in our study considered the frequency of changing bedsheets, we could not confirm results of a French study reporting an increased Der f 1 amount when changing bedsheets less often [59]. We found, however, slight positive correlations between household cleanliness and IgE levels to mite allergens. Analogously, subjects reporting to suffer from allergy lived in a cleaner household. The negative impact of extensive cleaning could relate to chemical cleaning agents, as e. g. propylene glycol and glycol ethers abundance in indoor air which was previously linked with allergic symptoms [60]. However, cleaning might also shift from very high to intermediate exposure levels and hence cause higher sensitization rates [40].

In conclusion, we could show that indoor allergen exposure can have various effects on IgE sensitization. The amount of allergens in homes is mainly influenced by pets, geographical regions and dwelling forms, while household cleanliness does not constitute a relevant parameter. Different exposure levels do not directly translate into IgE sensitization but are rather influenced by multiple parameters like farming environment or cat exposure which seemed to be general protective factors in our study. These findings were not strictly limited to indoor allergens as sensitization to pollen allergens was influenced by living forms or presence of pets (our unpublished data). In the present study, we concurrently investigated exposure and IgE levels to 5 representative purified indoor allergens in an epidemiological study cohort. Our results in combination with genetic predisposition and lifestyle will enable development of risk schemes for a more efficient management and potential prevention of allergic diseases.

## References

[1] Arshad SH. Does exposure to indoor allergens contribute to the development of asthma and allergy? Current allergy and asthma reports. 2010;10(1):49–55. Epub 2010/04/29. 10.1007/s11882-009-0082-6 20425514

[2] Platts-Mills TA, Vervloet D, Thomas WR, Aalberse RC, Chapman MD. Indoor allergens and asthma: report of the Third International Workshop. The Journal of allergy and clinical immunology. 1997;100(6 Pt 1):S2–24. Epub 1998/01/23. 9438476 10.1016/s0091-6749(97)70292-6

[3] Pomes A, Chapman MD, Wunschmann S. Indoor Allergens and Allergic Respiratory Disease. Curr Allergy Asthma Rep. 2016;16(6):43 10.1007/s11882-016-0622-9 27184001 PMC4976688

[4] Matricardi PM, Kleine-Tebbe J, Hoffmann HJ, Valenta R, Hilger C, Hofmaier S, et al EAACI Molecular Allergology User's Guide. Pediatr Allergy Immunol. 2016;27 Suppl 23:1–250. 27288833 10.1111/pai.12563

[5] van Ree R, van Leeuwen WA, Bulder I, Bond J, Aalberse RC. Purified natural and recombinant Fel d 1 and cat albumin in in vitro diagnostics for cat allergy. The Journal of allergy and clinical immunology. 1999;104(6):1223–30. Epub 1999/12/10. 10589005 10.1016/s0091-6749(99)70017-5

[6] Zahradnik E, Raulf M. Animal allergens and their presence in the environment. Front Immunol. 2014;5:76 10.3389/fimmu.2014.00076 24624129 PMC3939690

[7] Heinrich J, Bedada GB, Zock JP. Cat allergen level: its determinants and relationship to specific IgE to cat across European centers. Allergy Clin Immunol. 2006;118. 10.1016/j.jaci.2006.06.012 16950287

[8] Konieczny A, Morgenstern JP, Bizinkauskas CB, Lilley CH, Brauer AW, Bond JF, et al The major dog allergens, Can f 1 and Can f 2, are salivary lipocalin proteins: cloning and immunological characterization of the recombinant forms. Immunology. 1997;92(4):577–86. Epub 1998/03/14. 9497502 10.1046/j.1365-2567.1997.00386.x PMC1364166

[9] Salo PM, Yin M, Arbes SJ Jr., Cohn RD, Sever M, Muilenberg M, et al Dustborne Alternaria alternata antigens in US homes: results from the National Survey of Lead and Allergens in Housing. The Journal of allergy and clinical immunology. 2005;116(3):623–9. 10.1016/j.jaci.2005.05.030 16159634 PMC1635967

[10] Kleine-Tebbe J, Worm M, Jeep S, Matthiesen F, Lowenstein H, Kunkel G. Predominance of the major allergen (Alt a I) in Alternaria sensitized patients. Clinical and experimental allergy: journal of the British Society for Allergy and Clinical Immunology. 1993;23(3):211–8. Epub 1993/03/01. 7682472 10.1111/j.1365-2222.1993.tb00884.x

[11] Hirsch T, Stappenbeck C, Neumeister V, Weiland SK, Von Mutius E, Keil U, et al Exposure and allergic sensitization to cockroach allergen in East Germany. Clinical and experimental allergy: journal of the British Society for Allergy and Clinical Immunology. 2000;30(4):529–37. Epub 2000/03/16. 10718850 10.1046/j.1365-2222.2000.00785.x

[12] Arruda LK, Barbosa MC, Santos AB, Moreno AS, Chapman MD, Pomes A. Recombinant allergens for diagnosis of cockroach allergy. Curr Allergy Asthma Rep. 2014;14(4):428 10.1007/s11882-014-0428-6 24563284 PMC4179292

[13] Akdis CA, Agache I, editors. Global Atlas of Allergy: European Academy of Allergy and Clinical Immunology; 2014.

[14] Ownby DR, Johnson CC, Peterson EL. Exposure to dogs and cats in the first year of life and risk of allergic sensitization at 6 to 7 years of age. Jama. 2002;288(8):963–72. Epub 2002/08/23. 12190366 10.1001/jama.288.8.963

[15] Fujimura KE, Demoor T, Rauch M, Faruqi AA, Jang S, Johnson CC, et al House dust exposure mediates gut microbiome Lactobacillus enrichment and airway immune defense against allergens and virus infection. Proceedings of the National Academy of Sciences of the United States of America. 2014;111(2):805–10. Epub 2013/12/18. 10.1073/pnas.1310750111 24344318 PMC3896155

[16] von Mutius E. The microbial environment and its influence on asthma prevention in early life. The Journal of allergy and clinical immunology. 2016. Epub 2016/01/26. 10.1016/j.jaci.2015.12.1301 26806048

[17] Carlsten C, Dimich-Ward H, Becker AB, Ferguson A, Chan HW, DyBuncio A, et al Indoor allergen exposure, sensitization, and development of asthma in a high-risk birth cohort. Pediatric allergy and immunology: official publication of the European Society of Pediatric Allergy and Immunology. 2010;21(4 Pt 2):e740–6. Epub 2010/03/27. 20337962 10.1111/j.1399-3038.2010.01021.x

[18] Wahn U, Lau S, Bergmann R, Kulig M, Forster J, Bergmann K, et al Indoor allergen exposure is a risk factor for sensitization during the first three years of life. The Journal of allergy and clinical immunology. 1997;99(6 Pt 1):763–9. Epub 1997/06/01. 9215243 10.1016/s0091-6749(97)80009-7

[19] Olivieri M, Zock JP, Accordini S, Heinrich J, Jarvis D, Kunzli N, et al Risk factors for new-onset cat sensitization among adults: a population-based international cohort study. The Journal of allergy and clinical immunology. 2012;129(2):420–5. Epub 2011/12/16. 10.1016/j.jaci.2011.10.044 22168997

[20] Schoos AM, Chawes BL, Jelding-Dannemand E, Elfman LB, Bisgaard H. Early indoor aeroallergen exposure is not associated with development of sensitization or allergic rhinitis in high-risk children. Allergy. 2016;71(5):684–91. 10.1111/all.12853 26836471

[21] Wardzyńska A, Majkowska-Wojciechowska B, Pełka J, Korzon L, Kaczała M, Jarzębska M, et al Association of House Dust Allergen Concentrations With Residential Conditions in City and in Rural Houses. World Allergy Organization Journal. 2012;5(2):1–6. 23268467 10.1097/WOX.0b013e3182447fa8 PMC3488928

[22] Lee A, Sangsupawanich P, Ma S, Tan TN, Shek LP, Goh DL, et al Endotoxin levels in rural Thai and urban Singaporean homes. International archives of allergy and immunology. 2006;141(4):396–400. Epub 2006/09/01. 10.1159/000095467 16943679

[23] Gruchalla RS, Pongracic J, Plaut M, Evans R 3rd, Visness CM, Walter M, et al Inner City Asthma Study: relationships among sensitivity, allergen exposure, and asthma morbidity. The Journal of allergy and clinical immunology. 2005;115(3):478–85. Epub 2005/03/09. 10.1016/j.jaci.2004.12.006 15753892

[24] Grafetstatter C, Prossegger J, Braunschmid H, Sanovic R, Hahne P, Pichler C, et al No Concentration Decrease of House Dust Mite Allergens With Rising Altitude in Alpine Regions. Allergy, asthma & immunology research. 2016;8(4):312–8. Epub 2016/04/30. 10.4168/aair.2016.8.4.312 PMC4853508 27126724

[25] Chapman MD. Environmental allergen monitoring and control. Allergy. 1998;53(45 Suppl):48–53. Epub 1998/10/27. 9788707 10.1111/j.1398-9995.1998.tb04939.x

[26] Rao D, Phipatanakul W. Impact of environmental controls on childhood asthma. Current allergy and asthma reports. 2011;11(5):414–20. Epub 2011/06/29. 10.1007/s11882-011-0206-7 21710109 PMC3166452

[27] Stemeseder T, Klinglmayr E, Moser S, Lueftenegger L, Lang R, Himly M, et al Cross-sectional study on allergic sensitization of Austrian adolescents using molecule-based IgE profiling. Allergy. 2016. 10.1111/all.13071 27753449

[28] King EM, Filep S, Smith B, Platts-Mills T, Hamilton RG, Schmechel D, et al A multi-center ring trial of allergen analysis using fluorescent multiplex array technology. J Immunol Methods. 2013;387(1–2):89–95. 10.1016/j.jim.2012.09.015 23085532 PMC3955085

[29] R Core Team. R: A Language and Environment for Statistical Computing. Vienna, Austria: R Foundation for Statistical Computing; 2016.

[30] Custovic A, Taggart SC, Francis HC, Chapman MD, Woodcock A. Exposure to house dust mite allergens and the clinical activity of asthma. The Journal of allergy and clinical immunology. 1996;98(1):64–72. Epub 1996/07/01. 8765819 10.1016/s0091-6749(96)70227-0

[31] Hamilton RG. Assessment of indoor allergen exposure. Current allergy and asthma reports. 2005;5(5):394–401. Epub 2005/08/11. 16091213 10.1007/s11882-005-0013-0

[32] Simon-Nobbe B, Denk U, Poll V, Rid R, Breitenbach M. The spectrum of fungal allergy. Int Arch Allergy Immunol. 2008;145(1):58–86. 10.1159/000107578 17709917

[33] Salo PM, Arbes SJ Jr., Jaramillo R, Calatroni A, Weir CH, Sever ML, et al Prevalence of allergic sensitization in the United States: results from the National Health and Nutrition Examination Survey (NHANES) 2005–2006. The Journal of allergy and clinical immunology. 2014;134(2):350–9. Epub 2014/02/14. 10.1016/j.jaci.2013.12.1071 24522093 PMC4119838

[34] Bjerg A, Ekerljung L, Eriksson J, Naslund J, Sjolander S, Ronmark E, et al Increase in pollen sensitization in Swedish adults and protective effect of keeping animals in childhood. Clinical and experimental allergy: journal of the British Society for Allergy and Clinical Immunology. 2016. Epub 2016/05/10. 10.1111/cea.12757 27159904

[35] Asarnoj A, Hamsten C, Waden K, Lupinek C, Andersson N, Kull I, et al Sensitization to cat and dog allergen molecules in childhood and prediction of symptoms of cat and dog allergy in adolescence: A BAMSE/MeDALL study. J Allergy Clin Immunol. 2016;137(3):813–21 e7. 10.1016/j.jaci.2015.09.052 26686472 PMC6597346

[36] Perzanowski MS, Ronmark E, James HR, Hedman L, Schuyler AJ, Bjerg A, et al Relevance of specific IgE antibody titer to the prevalence, severity, and persistence of asthma among 19-year-olds in northern Sweden. J Allergy Clin Immunol. 2016. 10.1016/j.jaci.2016.05.017 PMC5405771 27430609

[37] Custovic A, Simpson BM, Simpson A, Hallam CL, Marolia H, Walsh D, et al Current mite, cat, and dog allergen exposure, pet ownership, and sensitization to inhalant allergens in adults. The Journal of allergy and clinical immunology. 2003;111(2):402–7. Epub 2003/02/18. 12589363 10.1067/mai.2003.55

[38] Radon K, Schottky A, Garz S, Koops F, Szadkowski D, Radon K, et al Distribution of dust-mite allergens (Lep d 2, Der p 1, Der f 1, Der 2) in pig-farming environments and sensitization of the respective farmers. Allergy. 2000;55(3):219–25. Epub 2001/02/07. 10753011 10.1034/j.1398-9995.2000.00461.x

[39] Lau S, Illi S, Sommerfeld C, Niggemann B, Bergmann R, von Mutius E, et al Early exposure to house-dust mite and cat allergens and development of childhood asthma: a cohort study. Multicentre Allergy Study Group. Lancet. 2000;356(9239):1392–7. Epub 2000/10/29. 11052581 10.1016/s0140-6736(00)02842-7

[40] Schram-Bijkerk D, Doekes G, Boeve M, Douwes J, Riedler J, Ublagger E, et al Nonlinear relations between house dust mite allergen levels and mite sensitization in farm and nonfarm children. Allergy. 2006;61(5):640–7. 10.1111/j.1398-9995.2006.01079.x 16629797

[41] Williams AH, Smith JT, Hudgens EE, Rhoney S, Ozkaynak H, Hamilton RG, et al Allergens in household dust and serological indicators of atopy and sensitization in Detroit children with history-based evidence of asthma. The Journal of asthma: official journal of the Association for the Care of Asthma. 2011;48(7):674–84. Epub 2011/08/11. 21827376 10.3109/02770903.2011.599909

[42] Vervloet D, Penaud A, Razzouk H, Senft M, Arnaud A, Boutin C, et al Altitude and house dust mites. The Journal of allergy and clinical immunology. 1982;69(3):290–6. Epub 1982/03/01. 7061770 10.1016/s0091-6749(82)80006-7

[43] Spieksma FT, Zuidema P, Leupen MJ. High altitude and house-dust mites. British medical journal. 1971;1(5740):82–4. Epub 1971/01/09. 5539181 10.1136/bmj.1.5740.82 PMC1795704

[44] Charpin D, Birnbaum J, Haddi E, Genard G, Lanteaume A, Toumi M, et al Altitude and allergy to house-dust mites. A paradigm of the influence of environmental exposure on allergic sensitization. The American review of respiratory disease. 1991;143(5 Pt 1):983–6. Epub 1991/05/01. 10.1164/ajrccm/143.5_Pt_1.983 2024854

[45] Ronmark E, Bjerg A, Perzanowski M, Platts-Mills T, Lundback B. Major increase in allergic sensitization in schoolchildren from 1996 to 2006 in northern Sweden. J Allergy Clin Immunol. 2009;124(2):357–63, 63 e1–15. 10.1016/j.jaci.2009.05.011 19577282 PMC2747664

[46] Valdivieso R, Iraloa V. Monthly variation of Dermatophagoides allergens and its influence on respiratory allergy in a high altitude environment (Quito, 2800 m a.s.l. in Andean Ecuador). Allergologia et immunopathologia. 2011;39(1):10–6. Epub 2010/09/21. 10.1016/j.aller.2010.02.010 20850216

[47] Elholm G, Linneberg A, Husemoen LL, Omland O, Gronager PM, Sigsgaard T, et al The Danish urban-rural gradient of allergic sensitization and disease in adults. Clinical and experimental allergy: journal of the British Society for Allergy and Clinical Immunology. 2016;46(1):103–11. Epub 2015/06/23. 26096697 10.1111/cea.12583

[48] Simons E, Curtin-Brosnan J, Buckley T, Breysse P, Eggleston PA. Indoor environmental differences between inner city and suburban homes of children with asthma. J Urban Health. 2007;84(4):577–90. 10.1007/s11524-007-9205-3 17551839 PMC2219555

[49] Perzanowski MS, Ronmark E, Platts-Mills TA, Lundback B. Effect of cat and dog ownership on sensitization and development of asthma among preteenage children. Am J Respir Crit Care Med. 2002;166(5):696–702. Epub 2002/09/03. 10.1164/rccm.2201035 12204868

[50] Majkowska-Wojciechowska B, Pełka J, Korzon L, Kozłowska A, Kaczała M. Prevalence of allergy, patterns of allergic sensitization and allergy risk factors in rural and urban children. Allergy. 2007;62. 10.1111/j.1398-9995.2007.01457.x 17686107

[51] Iversen M, Korsgaard J, Hallas T, Dahl R. Mite allergy and exposure to storage mites and house dust mites in farmers. Clinical and experimental allergy: journal of the British Society for Allergy and Clinical Immunology. 1990;20(2):211–9. Epub 1990/03/01. 2357620 10.1111/j.1365-2222.1990.tb02670.x

[52] Zahradnik E, Sander I, Kendzia B, Fleischer C, Bruning T, Raulf-Heimsoth M. Passive airborne dust sampling to assess mite antigen exposure in farming environments. J Environ Monit. 2011;13(9):2638–44. 10.1039/c1em10430f 21842065

[53] Sozanska B, Blaszczyk M, Pearce N, Cullinan P. Atopy and allergic respiratory disease in rural Poland before and after accession to the European Union. The Journal of allergy and clinical immunology. 2014;133(5):1347–53. Epub 2013/12/18. 10.1016/j.jaci.2013.10.035 24342546

[54] Horak E, Morass B, Ulmer H, Genuneit J, Braun-Fahrlander C, von Mutius E. Prevalence of wheezing and atopic diseases in Austrian schoolchildren in conjunction with urban, rural or farm residence. Wiener klinische Wochenschrift. 2014;126(17–18):532–6. Epub 2014/07/23. 10.1007/s00508-014-0571-z 25047409

[55] Ruokolainen L, von Hertzen L, Fyhrquist N, Laatikainen T, Lehtomaki J, Auvinen P, et al Green areas around homes reduce atopic sensitization in children. Allergy. 2014. Epub 2014/11/13. 10.1111/all.12545 PMC4303942 25388016

[56] Schoos AM, Chawes BL, Jelding-Dannemand E, Elfman LB, Bisgaard H. Early indoor aeroallergen exposure is not associated with development of sensitization or allergic rhinitis in high-risk children. Allergy. 2016. Epub 2016/02/03. 10.1111/all.12853 26836471

[57] Eller E, Roll S, Chen CM, Herbarth O, Wichmann HE, von Berg A, et al Meta-analysis of determinants for pet ownership in 12 European birth cohorts on asthma and allergies: a GA2LEN initiative. Allergy. 2008;63(11):1491–8. 10.1111/j.1398-9995.2008.01790.x 18721248

[58] Lau S, Illi S, Platts-Mills TA, Riposo D, Nickel R. Longitudinal study on the relationship between cat allergen and endotoxin exposure, sensitization, cat-specific IgG and development of asthma in childhood-report of the German Multicentre Allergy Study (MAS 90). Allergy. 2005;60. 10.1111/j.1398-9995.2005.00781.x 15876306

[59] Dallongeville A, Le Cann P, Zmirou-Navier D, Chevrier C, Costet N, Annesi-Maesano I, et al Concentration and determinants of molds and allergens in indoor air and house dust of French dwellings. Science of The Total Environment. 2015;536:964–72. 10.1016/j.scitotenv.2015.06.039. 26094801

[60] Choi H, Schmidbauer N, Sundell J, Hasselgren M, Spengler J, Bornehag CG. Common household chemicals and the allergy risks in pre-school age children. PloS one. 2010;5(10):e13423 Epub 2010/10/27. 10.1371/journal.pone.0013423 20976153 PMC2956675

